# Patient-centered activity monitoring in the self-management of chronic health conditions

**DOI:** 10.1186/s12916-015-0319-2

**Published:** 2015-04-09

**Authors:** Emil Chiauzzi, Carlos Rodarte, Pronabesh DasMahapatra

**Affiliations:** PatientsLikeMe, Inc., 155 Second Street, Cambridge, MA 02141 USA

**Keywords:** Activity monitoring, Chronic disease, Multiple sclerosis, Physical activity, Sensors, Wearables

## Abstract

**Background:**

As activity tracking devices become smaller, cheaper, and more consumer-accessible, they will be used more extensively across a wide variety of contexts. The expansion of activity tracking and personal data collection offers the potential for patient engagement in the management of chronic diseases. Consumer wearable devices for activity tracking have shown promise in post-surgery recovery in cardiac patients, pulmonary rehabilitation, and activity counseling in diabetic patients, among others. Unfortunately, the data generated by wearable devices is seldom integrated into programmatic self-management chronic disease regimens. In addition, there is lack of evidence supporting sustained use or effects on health outcomes, as studies have primarily focused on establishing the feasibility of monitoring activity and the association of measured activity with short-term benefits.

**Discussion:**

Monitoring devices can make a direct and real-time impact on self-management, but the validity and reliability of measurements need to be established. In order for patients to become engaged in wearable data gathering, key patient-centered issues relating to usefulness in care, motivation, the safety and privacy of information, and clinical integration need to be addressed. Because the successful usage of wearables requires an ability to comprehend and utilize personal health data, the user experience should account for individual differences in numeracy skills and apply evidence-based behavioral science principles to promote continued engagement.

**Summary:**

Activity monitoring has the potential to engage patients as advocates in their personalized care, as well as offer health care providers real world assessments of their patients’ daily activity patterns. This potential will be realized as the voice of the chronic disease patients is accounted for in the design of devices, measurements are validated against existing clinical assessments, devices become part of the treatment ‘prescription’, behavior change programs are used to engage patients in self-management, and best practices for clinical integration are defined.

## Background

### Activity monitoring by wearable device use in chronic disease management

Given what is known about the importance of tracking physical activity in the management of patients with chronic diseases, how can the current wave of consumer-accessible wearable technologies make the transition from personal wellness tools to patient-friendly clinical tools? A recent Pew Internet and American Life survey found that 69% of US adults track weight, diet, symptoms, or health routines in some manner [1]. Technological progress has fostered the development of a variety of ‘wearable’ devices and sensors for self-tracking health, including activity trackers, smart watches, smart clothing, patches and tattoos, ingestibles, and smart implants [2]. Historically, validated medical devices embedded with sensors have been applied in clinical trials and targeted research studies conducted in medical settings; however, advances in technology in activity (e.g., steps), physiological (e.g., blood oxygen saturation), and biochemical (e.g., pH) measurement have supported patient care and research outside of hospitals [3].

Currently, the most popular consumer-accessible wearable devices, e.g., Fitbit® and JawboneUP®, measure movements through accelerometers that apply algorithms to estimate activity levels (usually in the form of steps taken), sleep quality, and calories expended. Further, these wearable devices improve upon traditional pedometers as they include a number of additional behavior change techniques such as goal-setting, social support, social comparison, and rewards [4]. An Internet survey found that about 10% of Americans 18 years and over report ownership of a modern activity tracker such as a Fitbit® or JawboneUP® [5]. These wearable devices have been adopted by individuals seeking to enhance their personal fitness through increased personal health surveillance and social connections with others using the devices. Although the use of technology is often considered to be driven by younger age groups, the uptake of wearable devices is bimodally distributed – younger (25- to 34-year-old) groups use them for fitness enhancement, while older (55- to 64-year-old) groups use them to improve overall health [5]. Studies have shown the utility of activity monitoring in ostensibly healthy elderly or adult populations [6].

Can the use wearable devices lead to positive health impacts in chronic disease populations? There is some evidence to support this hypothesis. People with more serious health problems are more likely to report benefits as a result of tracking their health [1]. Activity monitors, with their ability to capture walking behavior in the real-world environment, may enable providers and patients to gain insights into the progression and impact of illnesses [7]. Further, as the population ages, there is increasing emphasis on reducing hospital stays, decrease readmission rates, and help patients manage their conditions in their home environments. Perhaps activity monitoring can be an effective tool in such disease self-management programs.

Available studies have primarily focused on establishing the feasibility of measuring activity and associating activity levels with beneficial outcomes. Activity-monitoring devices that record free-living walking behavior may be helpful in quantifying levels of ambulation and provide insights into important symptoms such as fatigue in multiple sclerosis patients [7,8]. The first study investigating the use of a modern wearable activity tracker on post-surgical mobility recovery during hospitalization was recently published by the Mayo Clinic [9]. This study found a significant relationship between the early recovery step count, hospital length of stay, and dismissal disposition in a group of elderly cardiac surgery patients. The use of accelerometers and pedometers to measure physical activity has also enhanced treatment for individuals participating in pulmonary rehabilitation [10]. A recent meta-analysis of activity monitor-based counseling studies with diabetes patients concluded that activity monitor-based counseling had a beneficial effect on physical activity, blood glucose, systolic blood pressure, and body mass index [11]. However, there is a lack of evidence demonstrating the effectiveness of activity monitors in chronic disease (e.g., osteoarthritis, cardiovascular diseases, type 2 diabetes mellitus, and chronic obstructive pulmonary disease) [12]. Studies have found modest short-term activity improvements and weight loss resulting from monitor and pedometer use [13-15], but it is not clear that these results are sustained over extended periods.

Self-management technology and physiological measurement have expanded beyond hospital settings to remote and direct use by patients with chronic diseases [16], but the adoption and utility of wearable devices for activity tracking is not well understood. Patients with chronic health conditions struggle with multifaceted needs that change during the course of their disease journeys. One path to success with wearable devices may reside in patient-driven health care, which encourages greater patient-physician collaboration, expanded patient social networks, and increased patient use of personal data for tracking their health outcomes [17]. In order to realize widespread adoption in a real-world community of chronic disease patients, key patient-centered questions need to be addressed, namely i) is the data useful in care? ii) how can users stay motivated to use devices? iii) does usage lead to behavior change and affect health outcomes? iv) is the information safe and will privacy be maintained? and v) will providers use this information in treatment planning and delivery? The promise of wearables will not be realized if they simply offer data – they must offer insights, outcomes, and engagement [18].

## Discussion

### Measurement and data validity

Good measurement properties of wearable device data are critical to successful adoption by patients and providers. The validity and reliability of measurements obtained from wearable devices and lack of standardization of devices continue to be of concern [19]. For example, it has been documented that the FitBit One® has good validity and reliability for step count, but not for distance travelled [20]. The Fitbit® and Fitbit Ultra® underestimate energy expenditure [21]. One study of eight different activity monitors (BodyMedia FIT®, Fitbit Zip®, Fitbit One®, Jawbone Up®, ActiGraph®, DirectLife®, NikeFuel Band®, and Basis B1 Band®) compared to a portable metabolic system (i.e., Oxycon Mobile®) found a mean percent error range from 9% to 24%, with BodyMedia FIT® on the low end and the Basis B1 Band® on the high end [22]. In addition, measurements are further confounded as wearable devices used for passive monitoring do not capture all free-living physical activity. For example, our observations of PatientsLikeMe (PLM) Fitbit® users found erroneous characterization of true activity due to infrequent or sporadic use and compromised accuracy due to predominant hand motion activities (playing the drums and cooking), driving or cycling, and long or short stride length. With technological advancement, it is expected that manufactures will respond to these limitations. Improved portability and ease of use, such as that offered in newly developed smart watches, could facilitate routine data gathering [23]. Physical activity estimations can be enhanced if there is an accounting of a range of indoor and outdoor activities, different walking speeds, and types of ambulation (walking, jogging, running) [24].

### User experience and engagement

In most instances, wearable devices are improving in terms of user experience, which includes extended battery life, easier synchronization via Bluetooth and WiFi, inclusion of additional sensor measures, and aesthetics from the wearable device itself and corresponding companion applications. However, the level of sustained use of wearable devices is ultimately dependent on the disease, patient behavior, and measurement need. One third of US owners of wearable devices stop using them within 6 months of first use [5].

Factors in long-term maintenance of wearable device usage include aesthetics, ease of set-up, lifestyle compatibility, and a clear value proposition to the user [5]. In a usability study of activity trackers, patients with chronic obstructive pulmonary disease were asked to rate 16 aspects of usability of both the Fitbit® and a wearable device called the Physical Activity Monitor [25]. The following concerns were identified by patients: i) technical difficulties (e.g., installation of device or software), ii) intention/willingness to monitor activity (e.g., willingness to use an activity monitor, recommend to others), iii) opinions towards wearing the device (e.g., pleasant, frightening, or frustrating to wear), and iv) the general attractiveness of the device. In contrast, the comfort in attaching or wearing the device, as well as the usefulness of activity monitoring, were rated high on the usability scale. It should be noted that some of these usability issues can be addressed by alterations to the devices (e.g., attention to fashion, good data visualization), whereas individual characteristics (e.g., negative expectations of use, inconsistent monitor use, lack of activity planning) may require a greater integration of engagement and behavior change principles.

In addition to usability factors, health literacy is of prime importance to maximize the benefit of consumer-accessible technology that may be utilized outside of clinical settings. According to the National Assessment of Adult Literacy [26], only 12% of US adults have proficient health literacy and over a third would have difficulty with following directions on a prescription drug label or maintaining a childhood immunization schedule based on a standard chart. In addition, limitations in numeracy, the ability to use numbers in daily living, are common in the US population [27]. These limitations may make it difficult to comprehend and utilize personal data. Will patients understand visualized data, attribute meaning to the ‘findings’, and make behavioral changes if necessary? Wearable devices capture potentially useful and clinically impactful data, but these devices will gain wider adoption when patients find devices usable and their data comprehensible.

### Behavior change

There is great variability in the effective use of behavioral science principles in order to effect behavior change. There are three key components that are critical to long-term engagement with wearables: i) habit formation (setting cues, routines, and rewards), ii) social motivation (sharing or competing for goals with others), and iii) goal reinforcement feedback to monitor personal progress [5]. BJ Fogg discusses the importance of computers as ‘persuasive technology’, with an ability to enhance self-efficacy, provide tailored information, trigger decision-making, and help people reduce barriers that impede target behaviors [28]. A recent review of behavioral techniques in activity monitors found that goal-setting, behavioral goal review, behavior feedback, self-monitoring, and rewards were generally included in popular monitors, but that other important components, such as problem-solving, behavioral instruction, and commitment strategies, were rare [4]. Overall, this review found that these devices included a range of 5 to 10 of 14 total potentially effective research-based techniques.

At present, the mechanisms of action are unclear. Do patients develop greater insight by viewing data about their activity levels? Does self-monitoring encourage a greater generalized sensitivity to one’s daily health status? Perhaps the act of tracking can itself lead to behavior change. It has long been known that simple self-monitoring can affect one’s self-evaluation and lead to self-administered consequences that affect response frequency [29]. Some wearable manufacturers attempt to leverage social media in the form of data sharing, but many consumers reject this idea [18]. The assumption that user measurement and feedback of data are sufficient in the success of these devices is dubious. The majority of consumer-accessible wearable devices are not part of a formal disease management program with a clearly reported time of use period and specific goals. The inclusion of effective behavior change components in a programmatic manner may drive positive outcomes more than wearable use alone [30].

### Privacy and safety

Many patients understand the value but state concerns about open-data sharing. Patients are increasingly amenable to sharing their data with peers [31]. PLM, in conjunction with the Institute of Medicine, conducted a survey of 2,125 PLM members and found that 94% of responders are willing to share their health information on social media if it helps doctors improve care [32]. In addition, 94% of responders would also be willing to share their health information on social media if it would help other patients like them, and 92% of responders would be willing to share information to help researchers learn more about their disease [32]. The 2014 State of the Internet of Things Study found that more than half of consumers are willing to share their wearable data with physicians [33].

On the other hand, a majority of those surveyed in the PLM/Institute of Medicine study agree that shared data can be used in negative ways [32]; 80% of consumers express privacy concerns about personal data-sharing [33]. This finding echoes a California Institute for Telecommunications and Information Technology survey, which found that 90% of respondents are seeking data anonymity [19]. These concerns are realistic, especially in an era of GPS technology, which could potentially reveal sensitive personal activities. Could such data open up health insurance subscribers with low activity levels to higher premiums?

Because of the issues related to the use and reuse of data in studies, the nature of informed consent is evolving [19]. Research suggests that individual openness to sharing will be dependent on the nature, use, user(s), legal protections, and potential compensation associated with the data [19,33]. Taken together, it is clear that greater transparency by device companies and researchers will be critical to patient engagement with these technologies.

### Care delivery and integration

Wearable adoption by patients in their health care will also be driven by their belief that such usage will have an impact on their care experience. How does the introduction of wearable devices that monitor activity impact treatment selection, treatment effectiveness, management of disease, and patient-doctor communication? Personal data collection offers the potential for greater patient-provider collaborations, but will require patient (and provider) confidence about the usefulness of the data in treatment planning. As seen with the rollout of electronic health records, providers may not welcome yet another data source due to concerns about additional pressures on their time, reimbursement, and workflow [34].

Wearable devices may provide insight into the progression and impact of illnesses and may provide insights with conditions in which activity levels or movement may be compromised, e.g., multiple sclerosis, depression, rheumatoid arthritis, pain, and chronic obstructive pulmonary disease. Does the use of this data affect the understanding of disease trajectory, symptom severity, and progression? Can this data ultimately affect disease-related quality of life and treatment effectiveness? Ultimately, patients and their providers will more likely consider wearable data if it impacts outcomes.

### The future

As physical activity tracking research evolves, the challenges in clinical measurement, adherence, privacy, and clinical integration need to be addressed before these devices are broadly adopted as clinical and self-management tools. The following research and clinical directions are being adopted by PLM and other organizations. First, the measurement need perceived by patients will define the creation and use of the appropriate wearable sensor systems. Rather than focus on retrofitting fitness wearables for medical applications, we believe that enabling patients to voice their needs will encourage the development of devices more attuned to chronic health needs. This will place greater emphasis on a user’s experience in tracking their chronic disease and potentially improve overall user engagement. Second, the data from wearable activity trackers will be increasingly understood in relation to established disease-specific clinical measures and assessments. For example, activity and mobility wearable data in multiple sclerosis will be understood in the context of measures such as the 6-Minute Walk Test [35]. This paves the way for provider acceptance of the data in clinical encounters. Device usage becomes part of the ‘prescription’ and patients share data as part of a treatment plan [36]. Third, as wearable sensors aim for increased sophistication, validation best practices will need to be established and new alliances will emerge. Project HoneyBee, for example, is evaluating the cost effectiveness of consumer physiological monitoring with heart disease, chronic obstructive pulmonary disease, atrial fibrillation, mobility, gait monitoring in hydrocephalus, and diabetes [37]. The Health Data Exploration project, with support from the Robert Wood Johnson Foundation [19], is bringing together researchers and industry to address key issues such as methodology, ethics, intellectual property, and intersections between data types. Fourth, wearables will be regarded as facilitators rather than drivers of change in health behavior [30]. The development of chronic disease behavior change programs that utilize activity and other measurements offer a more organized and engaging experience than use of the device alone.

## Summary

The ability to measure activity through a variety of methods will enable patients to assume greater control in their health care. Next generation wearables, multimodal smartphone technology, and ambient sensors (e.g., medical home) will increasingly enable real-time and continuous monitoring of activity. As more activity data is gathered in the context of disease progression and severity, the ability to use objectively captured data as early predictors of disease severity can have a profound impact in disease understanding and care delivery. However, key challenges in data validity, usability, programmatic integration, clinical integration, and user data privacy must be addressed. If these challenges can be navigated, there may substantial impact in chronic conditions that are characterized by activity limitations and corresponding functional impairments. In addition, meaningful and accurate activity measurements may represent an important endpoint in evaluations of treatment efficacy.

## Abbreviation

PLM: PatientsLikeMe
